# Communicating with African-American Women Who Have Had a Preterm Birth About Risks for Future Preterm Births

**DOI:** 10.1007/s40615-020-00697-8

**Published:** 2020-01-16

**Authors:** Allison S. Bryant, Laura E. Riley, Donna Neale, Washington Hill, Theodore B. Jones, Noelene K. Jeffers, Patricia O. Loftman, Camille A. Clare, Jennifer Gudeman

**Affiliations:** 1 Division of Maternal Fetal Medicine, Department of Obstetrics and Gynecology, Massachusetts General Hospital, 55 Fruit Street, Boston, MA 02114-2696 USA; 2 Department of Obstetrics and Gynecology, Weill Cornell Medicine, New York Presbyterian Hospital, 525 East 68th Street, New York, NY 10065 USA; 3 Division of Maternal-Fetal Medicine, The Johns Hopkins University, Johns Hopkins University School of Medicine, 600 North Wolfe Street, Baltimore, MD 21287-1228 USA; 4 Obstetrics, Gynecology, Maternal-Fetal Medicine, Florida Department of Health-Sarasota County, Sarasota Memorial Healthcare System, Center Place Health, 1750 17th Street, Building E, Sarasota, FL 34234 USA; 5 Department of Obstetrics and Gynecology, Wayne State University School of Medicine, Detroit, MI USA; 6 Oakwood Hospital – Dearborn, 18101 Oakwood Blvd, Suite 126, Dearborn, MI 48124 USA; 7 Johns Hopkins University School of Nursing, 525 North Wolfe Street, Baltimore, MD 21205 USA; 8 American College of Nurse-Midwives, New York, NY USA; 9 Department of Obstetrics and Gynecology, New York Medical College, Valhalla, NY USA; 10 New York City Health + Hospitals/Metropolitan, 1901 First Avenue Room 4B5, New York, NY 10029 USA; 11 Medical Affairs, AMAG Pharmaceuticals, Inc., 1100 Winter Street, Waltham, MA 02451 USA

**Keywords:** African-American women, Preterm birth, Maternal risk factors, Focus groups, Health inequities

## Abstract

**Purpose:**

African-American women are at higher risk of preterm birth (PTB) compared with other racial/ethnic groups in the USA. The primary objective was to evaluate the level of understanding among a group of African-American women concerning risks of PTB in future pregnancies. Secondary objectives were to evaluate how some women obtain information about PTB and to identify ways to raise their awareness.

**Methods:**

Six focus groups were conducted in three locations in the USA during 2016 with women (*N* = 60) who had experienced ≥ 1 PTB (< 37 weeks of gestation) during the last 5 years. The population was geographically, economically, and educationally diverse.

**Results:**

We observed a tendency to normalize PTB. Knowledge about potential complications for the infant was lacking and birth weight was prioritized over gestational age as an indicator of PTB. Participants were largely unaware of factors associated with increased PTB risk, such as a previous PTB and race/ethnicity. The most trusted information source was the obstetrical care provider, although participants reported relying on mobile apps, websites, and chat rooms. The optimal time to receive information about PTB risk in subsequent pregnancies was identified as the postpartum visit in the provider’s office.

**Conclusions:**

Awareness of the risks of recurrent PTB was limited in this diverse population. Educational programs on the late-stage development of neonates may strengthen knowledge on the relationship between gestational age and PTB and associated health/developmental implications. For educational efforts to be successful, a strong nonjudgmental, positive, solutions-oriented message focused on PTB risk factors is crucial.

## Introduction

The World Health Organization (WHO) defines preterm birth (PTB) as a live birth occurring before the completion of 37 weeks of gestation [1, 2]. The majority (84%) of PTBs occur after 32 weeks of gestation [3]. However, when compared with babies born at term, even babies born at 34–37 weeks have an increased risk of immediate complications, mortality, cerebral palsy [3], and developmental immaturity, which may have lifelong consequences [4, 5].

Spontaneous PTB is a multifactorial process resulting from the interplay of factors causing the uterus to change from a state of quiescence to one of active contractions, labor, and ultimately birth. The precursors vary by gestational age [6] and the precise cause of spontaneous preterm labor remains unidentified in approximately 50% of cases [7]. A higher risk of PTB among African-American women compared with White women in the USA is well established [8, 9]. The rate of PTB among African-American women in 2016 was about 50% higher than the rate among White women (14% vs. 9%) [10]. An increased risk for spontaneous PTB has also been observed in interracial/inter-ethnic couples where the woman is of African-American race [11]. Despite extensive documentation of racial/ethnic health inequities in the USA, the exact etiologies of the disparities remain unclear, though social determinants of health, structural racism, and disparities in access to care and quality of care play clear and critical roles in these etiologies [7, 12–17].

Clinicians representing the National Medical Association, the Society for Maternal-Fetal Medicine, and the American College of Nurse-Midwives met in March 2016 to discuss maternal and newborn health inequities related to PTB in the African-American community. Participants focused on reviewing key aspects of the issue and identifying solutions that the group, working collectively, could address. Here, we report the results of a series of focus groups designed and conducted to gain insight into the general awareness of the risk of a subsequent PTB among participating African-American women who had experienced a PTB. Secondary objectives were to evaluate how women obtain information about PTB and to identify potential additional ways to raise their awareness.

## Methods

Six focus groups were conducted in three US cities between October 4 and 12, 2016, with a total of 60 African-American women who had experienced ≥ 1 PTB in the last 5 years. Two focus groups were conducted in each location (19 participants in Atlanta, GA; 20 participants in Baltimore, MD; and 21 participants in Boston, MA); one group included women with a household annual income ≤ $49,999 and the second group included those with an annual income ≥ $50,000. It was hypothesized that women in low-income communities may have a lack of information.

Informed consent was obtained from all individual participants included in the study, which was conducted in accordance with prevailing ethical principles for market research. Participants were recruited and managed by focus group facilitators with robust experience in healthcare/medical industry research, using established practices for protecting the identity, information, and comfort of the participants. Women were invited from an opt-in panel based on their self-reported childbirth histories. Their anonymity was protected―identities were kept confidential from the researchers and sponsoring entity.

Quotes in the report are meant to be representative of the major themes drawn from the focus groups based upon analysis of their feedback. Several broad areas of discussion were set forth during the focus groups. The key themes and findings were identified based on the participant responses. In-person observation was conducted by professional market researchers who focused on verbal and non-verbal reactions, and a review of transcripts after the focus groups was performed to solidify understanding of what was said. With this information, the researchers analyzed major themes—rational and emotional—and the implications of those themes.

Additionally, the focus group guides were modified between each city to delve into themes seen in the previous groups. The objectives of the focus groups were to identify whether African-American women with prior PTBs (either spontaneous or medically indicated) were receiving information about future PTB risks; what messages they were receiving, who was delivering them and when they were being delivered; and whether there were different communication strategies that would resonate with women who were at high risk for PTB. This paper is not based upon clinical study or patient data.

## Results

### Demographics

The women in this study had a mean age of 33 years (range 20–45) and 53% had annual incomes ≤ $49,999. The majority of women (83%) had some college education, had graduated from college, or had an advanced degree. Sixty women gave birth to 71 children, 66 of whom were born preterm during the last 5 years. The most recent delivery for 72% of the women was within the last 2 years, and 70% of all most recent births occurred between 34 and 36 weeks of gestation (Table 1). The majority of women had singleton pregnancies, except for one woman who had twins.

**Table 1:** Demographic characteristics

Variables	Participants *N* = 60
Age in years	
Mean (range)	33 (20–45)
Household income, *n* (%)	
≤ $49,999	32 (53%)
≥ $50,000	28 (47%)
Education, *n* (%)	
High school/high school graduate	10 (17%)
Some college	22 (37%)
College graduate	21 (35%)
Graduate school/advanced degree	7 (12%)
Birth experience (most recent delivery), *n* (%)	
Gave birth within the last year	21 (35%)
Gave birth 1–2 years ago	22 (37%)
Gave birth 2–5 years ago	17 (28%)
Gestation age (most recent delivery), *n* (%)	
< 28 weeks	5 (8%)
28–33$$ \raisebox{1ex}{$6$}\!\left/ \!\raisebox{-1ex}{$7$}\right. $$weeks	12 (20%)
34–36$$ \raisebox{1ex}{$6$}\!\left/ \!\raisebox{-1ex}{$7$}\right. $$ weeks	42 (70%)
≥ 37 weeks	1 (2%)^a^

^a^In the Atlanta cohort, there was one woman who delivered between 0 and 12 months prior to the study at 37–38 weeks of gestation. She had previously delivered another child within 1–4 years prior to the study between 28 and 31 weeks of gestation

### Key Findings

We identified a lack of awareness about PTB through the group discussions. This lack of knowledge stemmed from 2 factors: (1) many of the women did not think that their previous PTB was abnormal and (2) these women were told very little about their risk for a future PTB. This was particularly true for women who had otherwise uncomplicated deliveries between 32 and 36 weeks, and whose babies were born close to perceived normal weight. Five key themes were identified:

#### A Lack of Knowledge About the Definition of PTB, Which Prevented the Women in this Study from Identifying That They Had, Indeed, Experienced One

Although all participants had at least one pregnancy in the last 5 years that resulted in a PTB at < 37 weeks, only approximately half considered their birth to be preterm. When asked what gestational age was considered premature, their answers ranged from 32 to 36 weeks. Relatively few women correctly identified a PTB as one that occurs at < 37 weeks of gestation. PTB was often incorrectly defined as one in which the baby was born underweight. If the baby was born at a normal weight and size, the women interviewed expressed less cause for concern.

I didn’t think my son was preterm because he was six or seven pounds. (woman with income $25–$49K; unknown education)

There was a tendency among the women to believe that there was a difference between the terms “preemie,” “preterm,” and “premature birth,” and especially between “preemie” and “preterm.” The women associated “preemie” with a more serious outcome, with the baby being underweight and likely needing to spend significant time in the neonatal intensive care unit (NICU), whereas “preterm” would signify only that the baby came early.

I think preemie is underweight and then preterm is, like, you know, early. (Baltimore woman with income $50–74K; post-graduate degree)

#### A Tendency to Normalize PTB

A family history of PTB sometimes normalized the construct. Some women assumed that if their own mothers gave birth at < 37 weeks, it must be a regular occurrence. Many believed that every birth is unique and thus, the experiences of other women would not necessarily have relevance for them; the closest attention was paid to their own mother’s experience due to the shared genetic background.

I guess you could trust your mom because I mean like she said, her mom had her at a young – prematurely so I guess you can – family history of having a baby early and stuff. (Atlanta woman with income $25–$49K; some college)

There was almost a fatalistic belief that nothing could be done to reduce the risk of PTB. Many of these women believed that each pregnancy was different for each woman and therefore, there was not much that they could do to change the outcomes.

I’m biased against [taking steps], because every pregnancy is different. (Baltimore woman with income < $25K; some college)

Everybody’s birth is different. (Boston woman with income $25–$49K, some college)

#### Varying Levels of Information About the Potential Long-term Health and Developmental Consequences of PTB

Women who had an uncomplicated pregnancy and/or delivery indicated that they received very little information about what to expect with their next pregnancy. However, women who had delivered extremely preterm or had complications, such as pre-eclampsia, generally had been counseled by their obstetrical (OB) care provider about potential complications with future pregnancies.

I feel like I needed more information about the bad stuff that could go wrong. (Atlanta woman with income < $25K; college degree)

I don't want my baby to be associated with behavioral problems because he was born early... but it’s something I needed to know. (Baltimore woman with income $50–70K; high school graduate)

#### A Lack of Knowledge Concerning Risk Factors for PTB

Most of the women had not heard or read anything about what to expect in future pregnancies. Furthermore, there was a difference in knowledge concerning the risks for PTB between women with an annual income ≤ $49,999 and women with an annual income ≥ $50,000. Only women who had experienced severe complications recalled specific conversations with their OB care provider about future risk for PTB.

We talked about the pregnancy, but I don’t think he talked about [what to expect for a future pregnancy]. (Boston woman with income $50–$74K; high school graduate)

Because I had [my son] at 31 weeks I would have liked to find out more about full-term pregnancy and I don't know any information pertaining to not doing that again. (Atlanta woman with income $25–$49; college degree)

#### A Desire to Receive Information About PTB from Their OB Care Provider (Preferably at the First Postpartum Visit)

Knowledge about pregnancy length, in general, tended to come from popular books, apps, or non-medical sources. The book, *What to Expect When You’re Expecting* [18], and the associated website (https://www.whattoexpect.com) and app were frequently referred to as sources of information. Other websites frequently mentioned as trusted and authoritative sources regarding pregnancy included https://www.babycenter.com and https://www.webmd.com. Although many women also cited other sources such as friends/family, chat boards, conventional wisdom, and even movies and TV shows, they tended to view these sources with a skepticism.

I’m always going to go to the doctor. I may run it by [my mom], like what do you think? Do you think I should be worried? But I’m still going to follow up. Even if I find something on the Internet – I may come to the doctor’s appointment with ‘this is what I researched,’ but I still want my doctor’s seal of approval. (Atlanta Mother, income and education unknown)

[Hospitals] could use a few [pamphlets] on babies born before 37 weeks. I had all the [hospital] stuff but I couldn't really use any of that information. (Atlanta Mother with income $25–49K, college graduate)

Women expressed an almost unanimous opinion that they would be unable to focus on anything related to their risk for a subsequent PTB during the hospital stay right after a PTB. The women in this study reported, however, that they did read the information in take-home packets that they received and would welcome more information on pregnancy length and PTB as part of those packages. OB care providers were viewed as trusted sources of medical information and the optimal messengers for information about pregnancy length. The women reported being most receptive to messages about pregnancy length during routine OB visits, such as the 6-week follow-up appointment in the controlled environment of the OB provider’s office or clinic. They also expressed a desire for messages to be nonjudgmental, positive, and solutions-oriented.

There are steps that can be taken to reduce [stress, smoking, health] … To just look at something and say, oh, diabetes, or African-American, [is unfair] because those things you can’t change. (Boston woman with income $75–$99K; college graduate)

## Discussion

While it has been established that women with an individual or family history of premature delivery are more likely to have a future PTB, many of the African-American women participating in our focus groups (70% of whom had singleton deliveries between 34 and 36 weeks) were unaware of the risks of PTB or even doubtful about whether they existed. The following five key themes were identified: There was (1) a lack of knowledge about the definition of PTB, which prevented the women in this study from identifying that they had, indeed, experienced one; (2) a tendency for the women to normalize PTB; (3) varying levels of information about the potential long-term health and developmental consequences of PTB; (4) a lack of knowledge concerning risk factors for PTB, and; (5) a desire to receive information about PTB from their OB care provider (preferably at the first postpartum visit). In addition, there was greater lack of information among lower income women (≤ $49,999) than higher income women (≥ $50,000), suggesting there needs to be a particular focus on providing education and information in all communities.

This report was not without its limitations. We know that the participants were cared for by a variety of OB providers but we did not specifically collect data on the type of OB care provider the women saw during their pregnancy (e.g., nurse-midwife, obstetrician-gynecologist specialist, maternal-fetal medicine subspecialist). We also did not explore the contribution of racial or cultural concordance between women and their providers; such concordance has been demonstrated to have a positive effect on communication and may influence adherence [19]. Women with both spontaneous and indicated PTBs were included in this survey, and we did not assess the role that the subtype of PTB may play in a woman’s experience, although we believe that messaging about prevention would be salient to all women at risk of PTB. Similarly, we were not able to fully explore if our findings were different among women based on gestational age at delivery, which would most likely have affected their experiences and perceptions about PTB.

The results of this research indicate that messages about PTB should clearly connect gestational age < 37 weeks to the terms preterm/premature/“preemie.” Discussions should be based on well-known, credible sources for health and risk information, and should emphasize that a full-term pregnancy is associated with significant benefits for the baby that go far beyond simple measurements of size or weight. We suggest an approach in which education about late stage in utero fetal development is coupled with specific complications that can occur when a baby is born preterm, such as difficulties with latching on and feeding or respiratory challenges. The risk of a subsequent PTB should be emphasized.

The OB care provider was viewed as a trusted source of medical information. The women in the study acknowledged lacking information about PTB. Beginning as early as the second trimester, women should begin to receive information from their OB care provider about PTB. Information can be transmitted in many forms such as linguistically and culturally appropriate booklets, handouts and flyers, mobile apps and websites, and should include definitions of preterm contractions and PTB; the difference between cramping and contractions and how to differentiate between the two; actions to take once the woman recognizes that she is having preterm contractions; and fetal growth and development at different gestational ages (explaining why it is beneficial to have the fetus remain in utero and explaining potential complications when the neonate is born too soon). Health-related messaging need not be restricted to healthcare environments, but can and should be effectively delivered in community settings as well.

Many of the women in this study reported being unaware that they were at risk of future PTBs and that the length of pregnancy carried relatively more influence than does birth weight. The statement that African-American women are an at-risk group was largely met with resentment by the women; this reaction seems valid and is meaningful. Evidence supports the premise that race itself is not the root cause of preterm birth, but that factors associated with being African-American—including experiencing institutional racism, racial health inequities, higher psychosocial stress, etc.—contribute to the increased rate of PTBs in African-American women [7, 12–17]. This recognition and context is important and related messaging should be delivered with these considerations, since women and their families will be understandably skeptical and/or dismissive of the notion that African-American race is itself is a driver of the occurrence of PTB. Simply presenting race as a risk factor without an appreciation of this broader context should be avoided.

This study found that when information was presented with a citation from a reputable and recognizable authority (e.g., the Centers for Disease Control and Prevention, or March of Dimes), the message was more appreciated. Although messages that focused on the negative aspects of PTB were of concern, some women in the group also expressed a need to know more about the risks of PTB, particularly if the risks are framed in a thoughtful, caring manner. The participants related that attention must be given to avoid utilizing a frightening and/or threatening tone. The women participating in these focus groups responded best to solutions-oriented messages as opposed to those that simply listed all negative outcomes. Specifically, the participants expressed that they would feel more optimistic and in control if they were armed a few concrete steps that they could take to reduce personal risks of future PTB. They described themselves as being motivated to talk about the risks with their OB care providers and to create a plan.

With the knowledge that there are important health benefits to a full-term birth and that mothers can play a vital role in the well-being of their babies, the women unanimously reported that they felt more agency over their situation. Correct implementation of an action-oriented plan using multiple channels of communication, particularly through mobile apps and websites, could help reinforce the message and ensure that it is delivered with a strong and lasting impact. Many women in the focus groups said that they would not be receptive to information about future pregnancies in the immediate postpartum period while still hospitalized, but they commented that the written materials from health care providers after discharge could be better utilized. Community home visiting programs, health centers, doulas, and community health workers could also be valuable adjuncts to communication.

The American College of Obstetricians and Gynecologists (ACOG) recently released a new paradigm for postpartum care aimed at reducing maternal morbidity and mortality [20], both of which are disproportionately represented among African-American women [21]. The organization recommends that postpartum care be viewed as an ongoing process tailored to each woman’s individual needs, and that all women should have contact with their OB care provider within 3 weeks postpartum versus the previous standard of 6 weeks. ACOG recommends that women whose pregnancies were complicated by PTB be counseled that they may be at increased risk for cardiometabolic disease, and that they be informed of the current recommendations to prevent a recurrent PTB [20].

A nonjudgmental, positive, solutions-oriented, patient-friendly approach regarding the risks of PTB could help women who are skeptical of the risks to internalize the message. The Health Belief Model (HBM) is one framework that may be utilized to address this messaging. Originated by social psychologists Hochbaum, Kegels, and Rosenstock in 1952 [22], this model addresses a lack of concern in preventive measures to address health and health outcomes. This theory was influenced by those of Kurt Lewin, who believed that “the world of the perceiver determines what an individual will and will not do” [23]. The HBM can predict health-related behaviors based on belief patterns. Therefore, if health beliefs may be changed, then behaviors may also be changed. Accordingly, six constructs predict health behavior: risk susceptibility, risk severity, benefits to action, barriers to action, self-efficacy, and cues to action [24].

The HBM states that one’s personal health behaviors may be affected by three factors: one’s general health values, specific health beliefs regarding one’s personal vulnerability to a health threat, and one’s beliefs in health consequences. The HBM can be applied to health education regarding PTB by providing women with incentives to learn more about risk factors of PTB, by providing action steps to mitigate those risks, and by enhancing their feelings of competency to prevent PTB. Other factors, however, can also influence one’s health behaviors and practices, including cultural factors, socioeconomic status, and previous experiences. As seen with the women in the six focus groups, compounding their lack of knowledge about risk factors for PTB, it was difficult to accept that being of the African-American race was also a perceived risk factor for PTB.

In conclusion, a lack of awareness may be preventing African-American women who have had a PTB from engaging in strategies designed to prevent subsequent PTBs. To counter the misconception that PTB is primarily about size and weight, OB care providers need to clearly emphasize the difference between gestational age and PTB. The authors suggest an approach in which education about late-stage fetal development in utero is coupled with information about the challenges for a neonate born too soon, including feeding and/or respiratory difficulties. Well known, credible sources for health and risk information are crucial. To successfully improve outcomes, information must be delivered via positive, solutions-oriented messages focused on solutions that every woman can do to lower their risk of future PTBs.

## References

[1] World Health Organization (1977). WHO: recommended definitions, terminology and format for statistical tables related to the perinatal period and use of a new certificate for cause of perinatal deaths. Modifications recommended by FIGO as amended October 14, 1976. Acta Obstet Gynecol Scand.

[2] World Health Organization. Born too soon: the global action report for preterm birth. In: Save the children, WHO. Geneva: World Health Organiatiion (WHO); 2012. http://www.who.int/pmnch/media/news/2012/preterm_birth_report/en/. Accessed 9 Sept 2019.

[3] Blencowe H, Cousens S, Oestergaard MZ, Chou D, Moller AB, Narwal R, Adler A, Vera Garcia C, Rohde S, Say L, Lawn JE (2012). National, regional, and worldwide estimates of preterm birth rates in the year 2010 with time trends since 1990 for selected countries: a systematic analysis and implications. Lancet..

[4] Mwaniki MK, Atieno M, Lawn JE, Newton CR (2012). Long-term neurodevelopmental outcomes after intrauterine and neonatal insults: a systematic review. Lancet..

[5] Teune MJ, Bakhuizen S, Gyamfi Bannerman C, Opmeer BC, van Kaam AH, van Wassenaer AG (2011). A systematic review of severe morbidity in infants born late preterm. Am J Obstet Gynecol.

[6] Steer P (2005). The epidemiology of preterm labour. Br J Obstet Gynaecol.

[7] Menon R (2008). Spontaneous preterm birth, a clinical dilemma: etiologic, pathophysiologic and genetic heterogeneities and racial disparity. Acta Obstet Gynecol Scand.

[8] McKinnon B, Yang S, Kramer MS, Bushnik T, Sheppard AJ, Kaufman JS (2016). Comparison of black-white disparities in preterm birth between Canada and the United States. Can Med Assoc J.

[9] Behrman RE, Butler AS (2007). Preterm birth: causes, consequences, and prevention.

[10] Centers for Disease Control and Prevention. Preterm Birth. https://www.cdc.gov/reproductivehealth/maternalinfanthealth/PretermBirth.htm. Accessed 26 Apr 2018.

[11] Shachar BZ, Mayo JA, Lyell DJ, Stevenson DK, Shaw GM, Blumenfeld YJ. Risk for spontaneous preterm birth among inter-racial/ethnic couples. J Matern Fetal Neonatal Med. 2017:1–7. 10.1080/14767058.2017.1293029. 10.1080/14767058.2017.1293029 28399669

[12] Bailey ZD, Krieger N, Agenor M, Graves J, Linos N, Bassett MT (2017). Structural racism and health inequities in the USA: evidence and interventions. Lancet..

[13] Braveman P, Heck K, Egerter S, Dominguez TP, Rinki C, Marchi KS, Curtis M (2017). Worry about racial discrimination: a missing piece of the puzzle of Black-White disparities in preterm birth?. PLoS One.

[14] Giurgescu C, Misra DP. Psychosocial factors and preterm birth among black mothers and fathers. MCN Am J Matern Child Nurs. 2018. 10.1097/nmc.0000000000000458. 10.1097/NMC.0000000000000458 29944478

[15] Grobman WA, Parker CB, Willinger M, Wing DA, Silver RM, Wapner RJ (2018). Racial disparities in adverse pregnancy outcomes and psychosocial stress. Obstet Gynecol.

[16] Manuck TA (2017). Racial and ethnic differences in preterm birth: a complex, multifactorial problem. Semin Perinatol.

[17] Wheeler S, Maxson P, Truong T, Swamy G (2018). Psychosocial stress and preterm birth: the impact of parity and race. Matern Child Health J.

[18] Murkoff H, Mazel S (2016). What to expect when you’re expecting.

[19] Alsan MGO, Graziani GC. Does diversity matter for health? Experimental evidence from Oakland. National Bureau of Economic Research. 2018;24787(4). 10.3386/w24787.

[20] American College of Obstetricians and Gynecologists Presidential Task Force on Redefining the Pospartum Visit (2018). ACOG Committee Opinion No. 736: Optimizing Postpartum Care. Obstet Gynecol.

[21] Jain JA, Temming LA, D’Alton ME, Gyamfi-Bannerman C, Tuuli M, Louis JM, Srinivas SK, Caughey AB, Grobman WA, Hehir M, Howell E, Saade GR, Tita ATN, Riley LE (2018). SMFM special report: putting the "M" back in MFM: reducing racial and ethnic disparities in maternal morbidity and mortality: a call to action. Am J Obstet Gynecol.

[22] Hochbaum G, Rosenstock I, Kegeles S. Health Belief Model. 1952. https://www.utwente.nl/en/bms/communication-theories/sorted-by-cluster/Health%20Communication/Health_Belief_Model/. Accessed 9 Sept 2019.

[23] O’Dea N, Saltman DD, Editors. Health beliefs. Oxford textbook of primary medical care. New York: Oxford University Press; 2005.

[24] Jones CL, Jensen JD, Scherr CL, Brown NR, Christy K, Weaver J (2015). The Health Belief Model as an explanatory framework in communication research: exploring parallel, serial, and moderated mediation. Health Commun.

